# Anaesthesia by intravenous propofol reduces the incidence of intra-operative gastric electrical slow-wave dysrhythmias compared to isoflurane

**DOI:** 10.1038/s41598-023-38612-w

**Published:** 2023-07-21

**Authors:** Zahra Aghababaie, Tim Hsu-Han Wang, Linley A. Nisbet, Ashton Matthee, Jarrah Dowrick, Gregory B. Sands, Niranchan Paskaranandavadivel, Leo K. Cheng, Gregory O’Grady, Timothy R. Angeli-Gordon

**Affiliations:** 1 Auckland Bioengineering Institute, University of Auckland, Private Bag 92019, Auckland, New Zealand; 2 Department of Surgery, University of Auckland, Auckland, New Zealand

**Keywords:** Gastroenterology, Medical research, Biomarkers, Preclinical research, Biomedical engineering

## Abstract

Gastric motility is coordinated by bioelectrical slow-wave activity, and abnormal electrical dysrhythmias have been associated with nausea and vomiting. Studies have often been conducted under general anaesthesia, while the impact of general anaesthesia on slow-wave activity has not been studied. Clinical studies have shown that propofol anaesthesia reduces postoperative nausea and vomiting (PONV) compared with isoflurane, while the underlying mechanisms remain unclear. In this study, we investigated the effects of two anaesthetic drugs, intravenous (IV) propofol and volatile isoflurane, on slow-wave activity. In vivo experiments were performed in female weaner pigs (*n* = 24). Zolazepam and tiletamine were used to induce general anaesthesia, which was maintained using either IV propofol (*n* = 12) or isoflurane (*n* = 12). High-resolution electrical mapping of slow-wave activity was performed. Slow-wave dysrhythmias occurred less often in the propofol group, both in the duration of the recorded period that was dysrhythmic (propofol 14 ± 26%, isoflurane 43 ± 39%, *P* = 0.043 (Mann–Whitney* U* test)), and in a case-by-case basis (propofol 3/12, isoflurane 8/12, *P* = 0.015 (Chi-squared test)). Slow-wave amplitude was similar, while velocity and frequency were higher in the propofol group than the isoflurane group (*P* < 0.001 (Student’s *t-*test)). This study presents a potential physiological biomarker linked to recent observations of reduced PONV with IV propofol. The results suggest that propofol is a more suitable anaesthetic for studying slow-wave patterns in vivo.

## Introduction

Gastric motility is coordinated, in part, by underlying electrical activity known as slow waves. In the normal stomach, slow waves initiate from a dominant pacemaker site located on the upper corpus on the greater curvature and propagate distally to the terminal antrum^1,2^. Abnormal ‘dysrhythmic’ slow-wave activity has been implicated in several motility disorders, including gastroparesis^3^, and chronic nausea and vomiting^4,5^, fuelling clinical and research interest in these dysrhythmias^6,7^.

In the past two decades, technological advancement has provided high-resolution mapping of gastrointestinal (GI) slow-wave activity^8^. Recording and analysis of slow-wave activity, particularly in high-resolution^4,9^, offers a diagnostic biomarker for GI dysfunction where new biomarkers are critically needed^6,10^. Slow-wave mapping also underpins the development of potential treatments to modulate slow-wave abnormalities, such as gastric ablation^11,12^, and pacing^13,14^.

Invasive high-resolution gastric mapping is typically performed in the anaesthetised state. Despite this, the influence of anaesthetic agents on slow-wave activity, and the possibility of anaesthesia-induced dysrhythmias, remain uncertain. Preliminary studies have shown that gastric and intestinal slow waves show impaired activity following isoflurane anaesthesia^15–17^. In addition, anaesthesia with isoflurane resulted in decreased gastric myoelectric signal power compared to the awake state^16^. A comparison of gaseous isoflurane and intraperitoneal injection of thiobutabarbital in a rodent model showed that isoflurane resulted in reduced motility^18^. However, the effects of alternative intravenous (IV) agents on gastric slow waves, such as propofol, have not been evaluated.

Clinical studies have shown that IV propofol reduces the incidence of postoperative nausea and vomiting (PONV) compared with isoflurane anaesthesia^19,20^. As a result, and due to potential immunosuppression advantages^21^, a shift has been advocated for anaesthesia choices towards total IV anaesthesia (TIVA) using propofol and away from using volatile anaesthetics like isoflurane. However, the physiological mechanisms underlying the reduction of PONV associated with propofol anaesthesia have yet to be established^21^. Since slow-wave dysrhythmias have been associated with nausea and vomiting^4,6,7^, it may be hypothesised that anaesthesia-induced dysrhythmias could be a contributing factor to post-surgical dysmotility.

In this study, we aimed to investigate the effect of two widely-used anaesthetic drugs (propofol and isoflurane) on slow-wave activity and compare their impact on gastric slow-wave activity.

## Results

This study comprised a total of 24 animals, with a mean weight of 34.8 ± 4.1 kg. The dataset comprised a total of 200 min of high-resolution electrical mapping, with a mean duration of 8.3 ± 3.0 min per experiment (propofol group 8.1 ± 2.5 min, isoflurane group 8.5 ± 3.6 min, *P* = 0.407).

### Occurrence of Slow-Wave Dysrhythmias

The overall occurrence of dysrhythmias in the propofol group was significantly lower than that of the isoflurane group (propofol: 14 ± 26% of the total recorded duration, median = 0, Q1 = 0, Q3 = 8%; isoflurane: 43 ± 39% of the total recorded duration, median = 33%, Q1 = 0, Q3 = 73%; *P* = 0.043 (Mann–Whitney* U* test)).

**Figure 1:**
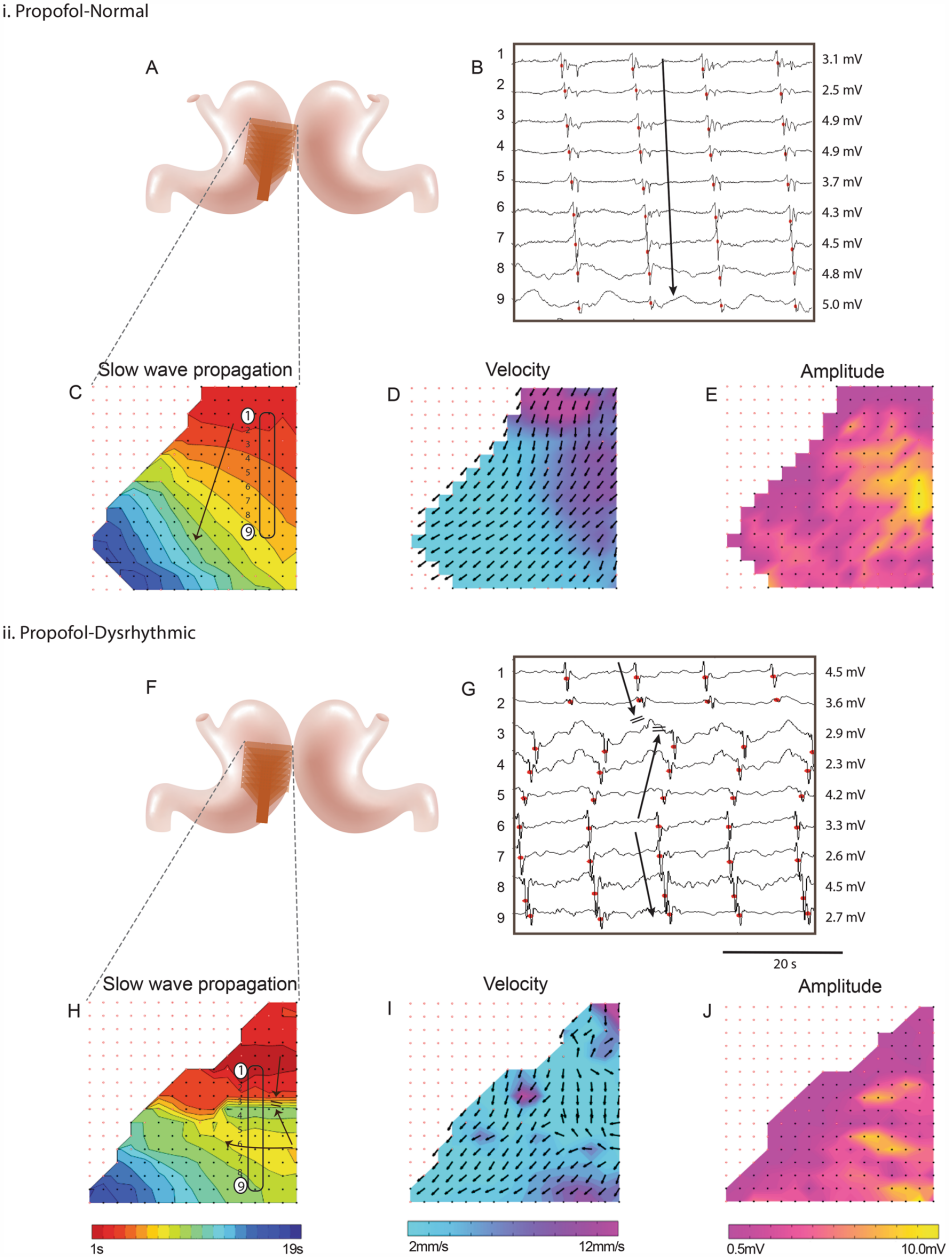
Representative high-resolution slow-wave mapping results of: **i.** Normal antegrade propagation in a female pig anaesthetised with intravenous (IV) propofol; **ii.** Dysrhythmic propagation in a female pig anaesthetised with IV propofol. (**A**,**F**) Position of the flexible-printed-circuit (FPC) electrode array on the stomach. (**B**,**G**) Electrograms from the 8 electrode positions labelled in (**C**,**H**), with slow-wave activation times (AT) marked as red dots. (**C**,**H**) Isochronal AT map of slow-wave propagation. Each colour band indicates the area of slow-wave propagation per 1 s from red (early) to blue (late). (**D**,**I**) Velocity map of the same slow wave, showing the speed (colour spectrum) and direction (arrows) of the wave at each point on the array. (**E**,**J**) Amplitude map of the same wave.

For cases anaesthetised with propofol, 75% (9/12) demonstrated consistently normal antegrade slow-wave propagation (Table 1, Fig. 1i). The other 25% of cases (3/12) demonstrated dysrhythmic activity for at least a portion of the recording period (Fig. 1ii).

**Table 1:** Comparison of propofol and isoflurane anaesthesia on gastric slow-wave patterns.

	Propofol group	Isoflurane group
*n*	12	12
Induction anaesthetic	Zolazepam / tiletamine (0.1 ml/kg)	Zolazepam / tiletamine (0.1 ml/kg)
Maintenance anaesthetic	IV propofol (Diprivan 2%, 0.2–0.4 mg/kg/min)	Volatile isoflurane (2.5–5%, oxygen flow of 400 mL/min)
Duration of recording (min)	8.1 ± 3	8.5 ± 4
Dysrhythmia occurrence mean ± SD	14 ± 26% *	43 ± 39% *
Dysrhythmia occurrence Q1, median, Q3	0, 0, 8% *	0, 33, 73% *
Cases with dysrhythmic propagation	25% (3/12 cases) **	66% (8/12 cases) **

*P = 0.043 (Mann–Whitney *U* test).

**P = 0.015 (Chi-squared test).

**Figure 2:**
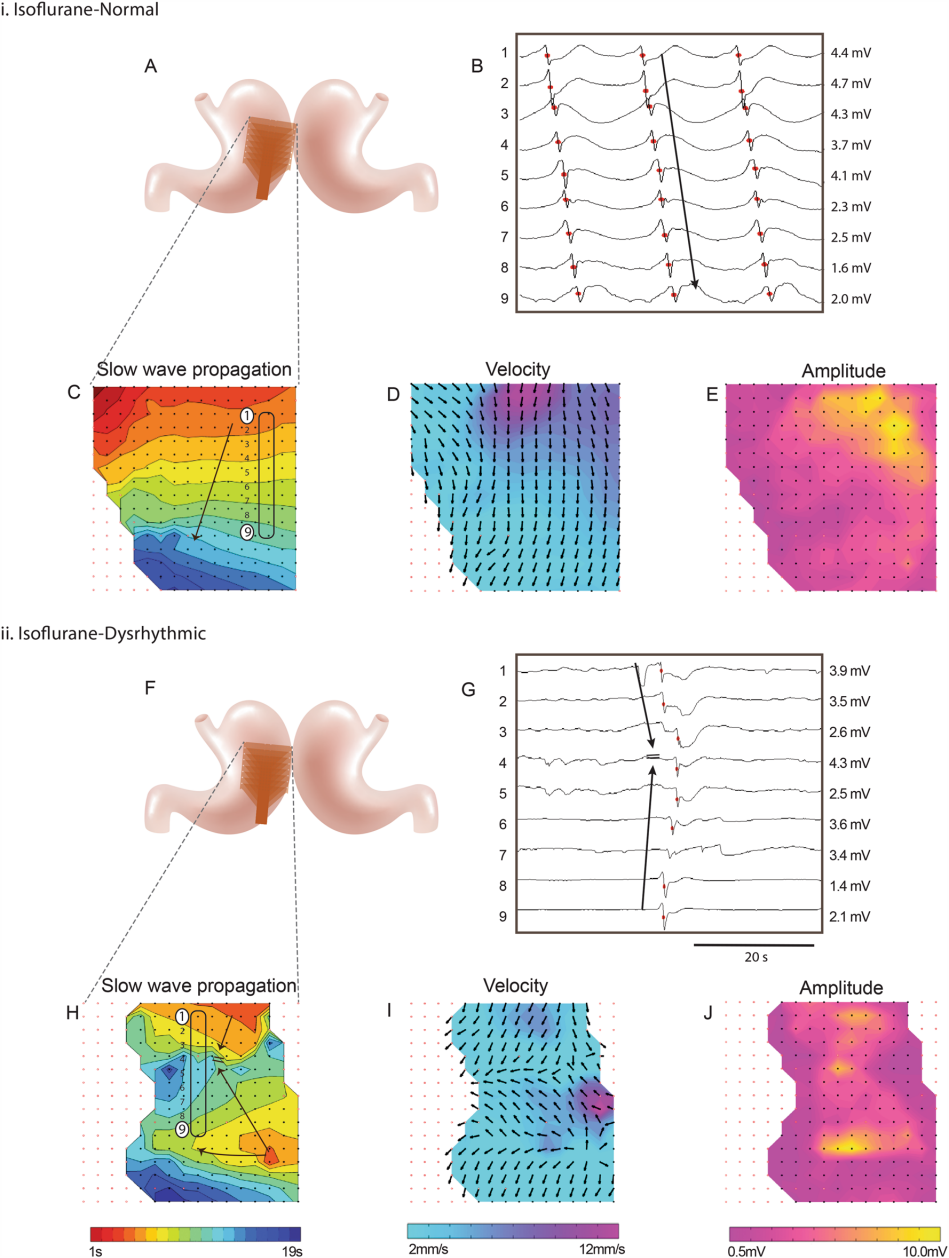
Representative high-resolution slow-wave mapping results of: **i**. Normal antegrade propagation in a female pig anaesthetised with volatile isoflurane; **ii.** Dysrhythmic propagation in a female pig anaesthetised with volatile isoflurane. (**A**,**F**) Position of the flexible-printed-circuit (FPC) electrode array on the stomach. (**B**,**G**) Electrograms from the 8 electrode positions labelled in (**C**,**H**), with slow-wave activation times (AT) marked as red dots. (**C**,**H**) Isochronal AT map of slow-wave propagation. Each colour band indicates the area of slow-wave propagation per 1 s from red (early) to blue (late). (**D**,**I**) Velocity map of the same slow wave, showing the speed (colour spectrum) and direction (arrows) of the wave at each point on the array. (**E**,**J**) Amplitude map of the same wave.

In contrast, consistent normal antegrade slow-wave propagation was observed in 33% (4/12) of cases anaesthetised with isoflurane (Table 1, Fig. 2i). The other 67% of cases (8/12) demonstrated dysrhythmic activity for at least a portion of the recording period (Fig. 2ii) (propofol: 3/12 cases demonstrated dysrhythmic activity; isoflurane: 8/12 cases demonstrated dysrhythmic activity; *P* = 0.015 (Chi-squared test)).

### Slow-wave Characteristics

**Figure 3:**
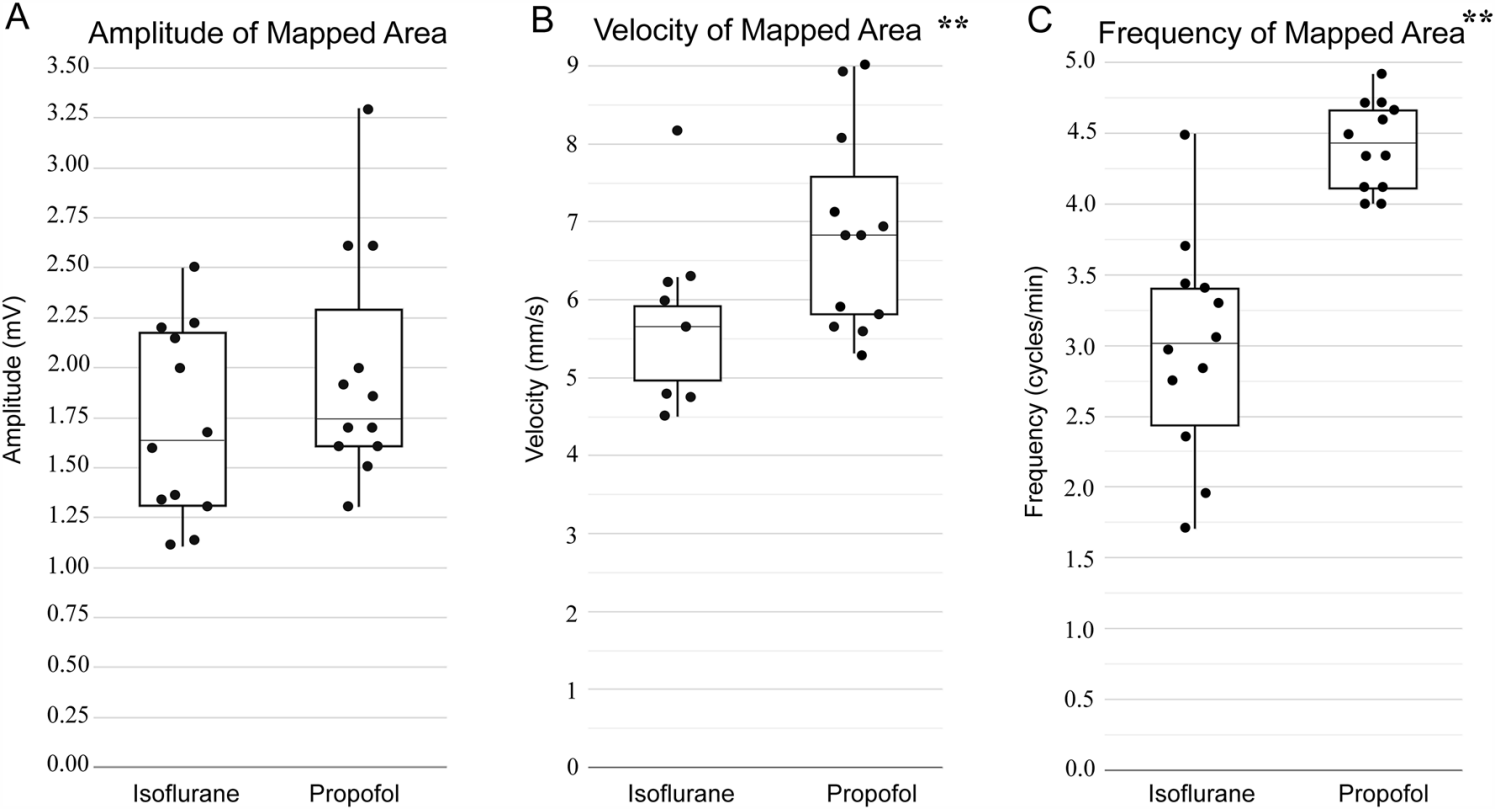
Slow-wave characteristics from the in vivo stomach in female pigs anaesthetised by volatile isoflurane (*n* = 12) versus intravenous (IV) propofol (*n* = 12). (**A**) Slow-wave amplitude was similar between isoflurane and propofol. (**B**) Slow-wave velocity was higher with propofol compared to isoflurane. (**C**) Slow-wave frequency was also higher with propofol compared to isoflurane. **denotes statistical significance of *P* < 0.05 (Student’s *t-*test).

Slow-wave amplitude was similar in the propofol and isoflurane groups (propofol: 2.0 ± 0.6 mV vs isoflurane: 1.7 ± 0.5 mV; *P* = 0.339 (Mann–Whitney* U* test)) (Fig. 3A). Slow-wave velocity was significantly higher in the propofol group compared to the isoflurane group (propofol: 6.8 ± 1.2 mm/s vs isoflurane: 5.7 ± 0.8 mm/s; *P* = 0.008 (Student’s *t-*test)) (Fig. 3B). Slow-wave frequency was also significantly higher in the propofol group compared with the isoflurane group (propofol: 4.4 ± 0.3 cpm vs isoflurane: 3.0 ± 0.8 cpm; *P* < 0.001 (Student’s *t-*test)) (Fig. 3C).

## Discussion

In this study, we investigated the impact of anaesthetic agents on gastric slow-wave activity. Slow-wave activity in cases anaesthetised with propofol showed significantly less dysrhythmic activity compared to cases anaesthetised with isoflurane (dysrhythmic occurrence of 14 ± 26% vs 43 ± 39%, respectively). In addition, slow-wave activity had significantly higher frequency in the propofol group with lower standard deviation, suggesting increased stability of slow-wave activity with propofol. These results provide a potential physiological biomarker that may help to explain clinical observations of decreased PONV with propofol.

PONV is a common problem encountered in patients following administration of general anaesthesia^19,20,22^. Traditionally, general anaesthesia is induced and maintained by a combination of volatile and IV agents. However, there is now a trend towards TIVA, supported by findings that TIVA results in reduced rates of PONV, along with other benefits including faster emergence from anaesthesia and reduced incidence of post-operative delirium in elderly patients^23–25^. The mechanisms underlying the observed reduction in PONV remain unclear. Our present study now introduces high-resolution gastric mapping techniques to assess the impact of volatile versus IV anaesthesia. We identified a reduction in gastric electrical dysrhythmias in pigs anaesthetised with propofol compared to isoflurane. The exact mechanisms underlying the increase of dysrhythmia with isoflurane are currently unknown. Potential mechanisms include suppression of ICC entrainment, suppressed vagal activity^26^, and/or changes to splanchnic blood flow and oxygenation^27,28^, but future studies are required to identify the underlying mechanisms. Pigs are a common and validated model for gastric physiology^8^, and gastric electrical dysrhythmias have been associated with nausea and vomiting in humans^4,6,29^. The findings from this present study introduce gastric dysrhythmias as a physiological biomarker that is consistent with clinical observations of the reduction in PONV incidence following the use of TIVA, therefore providing preliminary evidence that further supports a shift towards TIVA in general anaesthesia.

Our results of reduced dysrhythmias and less variable slow-wave frequency under propofol suggest that propofol is a better anaesthetic option for recording and studying normal rhythmic slow-wave activity. This finding may be particularly relevant for future applications aiming to use slow-wave recordings in human patients for diagnostic and treatment purposes^30^. However, the higher frequency of slow waves under propofol, compared to EGG results in the porcine model, suggest that propofol may also have an impact on frequency of slow waves^31^. The reduced incidence of dysrhythmia with propofol is beneficial because inducing dysrhythmia with the anaesthetic agent could impact the diagnostic outcome and subsequently negatively impact potential treatments to eliminate dysrhythmias, such as gastric ablation or gastric pacing^11,13^. On the contrary, a higher incidence of dysrhythmia when using isoflurane offers a potentially valuable tool for investigators wanting to study abnormal slow-wave activity in research applications, for example, when investigating the feasibility and efficacy of new techniques for detecting or eliminating dysrhythmias^29^. Our results of dysrhythmia occurrence, frequency, amplitude, and velocity under isoflurane agree with previous studies in the porcine model^11,31^. In addition, a study using computed-tomography (CT) imaging in a rodent model has shown that isoflurane anaesthesia decreased gastric emptying compared to the non-anaesthetised state^32^, aligning with our findings of decreased slow-wave frequency with isoflurane.

This study was limited to relatively short-duration recordings of intraoperative slow-wave activity. It has previously been observed that slow-wave stability deteriorates under prolonged anaesthesia by isoflurane^33^, and we anecdotally observed that slow-wave frequency remained more consistent under propofol, including late in the recording periods^34^. However, this observation requires verification. To date, human gastric-mapping studies have shown relatively normal gastric slow-wave activity in healthy controls, however most of these studies were performed immediately after induction and laparotomy^4,29^. Future studies of longer duration recordings, including in the post-operative period, are necessary to understand the full impact of anaesthetic agents during and after surgery. In addition, the slow-wave recording methods in this study were surgically invasive. While these direct-contact measurements enabled accurate dysrhythmia detection, measuring the slow-wave activity of the stomach with less invasive methods, such as body surface^29^ and/or endoscopic mapping^35,36^, with accompanying continuous measurement of nausea severity^6^, would enable the evaluation of gastric slow wave rhythms without surgical intervention. Such approaches would enable the translation of these methods to patients, where the potential role and clinical impact of anaesthesia and dysrhythmias in PONV and post-surgical dysmotility can be investigated.

## Methods

Ethical approval was granted by the University of Auckland Animal Ethics Committee (AEC3090 and AEC8158), and all methods were performed in accordance with the relevant guidelines and regulations. The study is reported in accordance with the ARRIVE guidelines.

All experiments were performed in vivo on female crossbreed weaner pigs fasted overnight. All animals were subjected to general anaesthesia with zolazepam and tiletamine (0.1 ml/kg, Zoletil, Virbac, NZ). The first group of animals (propofol group, *n* = 12) was maintained under anaesthesia with IV propofol (Diprivan 2%, 0.2–0.4 mg/kg/min, AstraZeneca, UK), and the second group of animals (isoflurane group, *n* = 12) were maintained under anaesthesia with gaseous isoflurane (2.5–5% with an oxygen flow of 400 mL/min within a closed-circuit anaesthetic system).

Vital signs, including heart rate, blood pressure, and rectal temperature, were monitored and maintained within the normal range. A midline laparotomy (10 cm) was performed, and the gastric serosal surface was exposed to enable high-resolution mapping. The subjects of the propofol group were also part of a separate recovery study^34^, and subjects of the isoflurane group were part of separate acute studies^11,12,37^. Recordings were consistently performed at the beginning of the initial surgical period in both cohorts prior to any intervention and surgical methods were consistent, except that the propofol group received prophylactic antibiotics by injection immediately prior to the surgery (200 mg/ml, 5 mg/kg, Ceftiofur, Zoetis, NZ). At the conclusion of the experiments, the animals were euthanised with a lethal bolus injection of sodium pentobarbital while still under anaesthesia.

### High-resolution mapping

Intraoperative high-resolution electrical mapping was performed using validated flexible-printed-circuit (FPC) electrode arrays (256 electrodes, 16 × 16 array, 4 mm spacing; FlexiMap, NZ)^8,9^. The FPC array was gently positioned over the corpus and overlain with warm (37 °C) saline-soaked gauze to maintain moisture and gentle pressure of the electrodes onto the serosa. The wound edges were approximated with surgical clamps.

### Electrophysiological signal acquisition and analysis

Bioelectrical signals were acquired at 512 Hz using a passive recording system (ActiveTwo, BioSemi, Netherlands). Signal processing and analysis was performed in the Gastrointestinal Electrical Mapping Suite (FlexiMap, Auckland, NZ)^38^. Data were first down-sampled to 30 Hz before baseline drift was estimated and removed using a Gaussian moving median filter. A Savitzky-Golay filter (‘low-pass’, ~ 2 Hz) was then applied to reduce high-frequency noise^39^. Slow-wave activation times (AT) were marked and clustered^38^. Slow-wave propagation was visualised using isochronal AT maps showing the area of propagation per unit of time (Fig. 1)^38^. Slow-wave amplitude, velocity, and frequency were calculated and mapped.

The occurrence of dysrhythmic activity was calculated as the duration of dysrhythmic activity (e.g., ectopic pacemakers, retrograde propagation, colliding wave fronts, conduction blocks, or electrical quiescence)^4,29^ divided by the total recorded duration. Each classification of normal versus dysrhythmic activity was subsequently verified by at least three other experienced investigators.

### Statistical analysis

Quantitative data were presented as median, quartiles (Q1 and Q3) and mean ± standard deviation. A normality test (Shapiro–Wilk test) was performed on quantitative data. Based on the result of the normality test, statistical differences were compared using paired Student’s *t*-test for data that follow a normal distribution, and Mann–Whitney *U* test for data for data that does not follow a normal distribution. Statistical differences in a case-by-case basis were compared using Chi-squared test. The significance threshold was *P* < 0.05.

## Data Availability

The datasets generated during and/or analysed during the current study are available from the corresponding author on reasonable request.
